# Management of Gastric Variceal Bleed by Endoscopic Cyanoacrylate Injection: A Case Series

**DOI:** 10.7759/cureus.70836

**Published:** 2024-10-04

**Authors:** Ajay Patwa, Virendra Atam, M. L. Patel, Faraz Ahmad, K. K Gupta, Harish Gupta, Satish Kumar, Archana Devi, Priya Mishra, Anurag Chaudhary

**Affiliations:** 1 Medicine, Gastroenterology and Hepatology, King George’s Medical University, Lucknow, IND; 2 Internal Medicine, King George’s Medical University, Lucknow, India., Lucknow, IND; 3 Medicine, King George's Medical University, Lucknow, IND; 4 Surgery, King George's Medical University, Lucknow, IND; 5 Gastroenterology and Hepatology, King George's Medical University, Lucknow, IND; 6 Internal Medicine, King George's Medical University, Lucknow, IND

**Keywords:** cyanoacrylate glue, endotherapy, gastric varices, hemostasis, upper gastrointestinal bleed

## Abstract

Background and aims

Cyanoacrylate glue (CAG) is the standard of care for gastric varices (GVs) but has serious complications too. The literature is scarce on determining the safe and effective amount of glue before the procedure objectively. Our study aimed to fill this gap.

Methods

It was an interventional case series, from January to December 2022. Patients with GVs, in whom CAG was injected, were included. Demographic, clinical, and endoscopic data with emphasis on cumulative variceal diameter (CVD, sum of maximum diameter of each varix), the total amount of glue injected (G_Total_), outcomes (technical and clinical success), and complications intra- and post-procedural) were noted.

Results

Among 467, 18 (4%) patients had gastric varices. Glue was injected in six (1%) patients. Five had type 2 gastro-esophageal varices (GOV2) and one had type 1 isolated gastric varix (IGV1). Four had a history of upper GI bleed. Numbers of GVs ranged between 1 and 4, sizes 0.5-2.5 cm, and CVDs between 3.5 and 5.0 cm. G_Total_ ranged between 2 and 4 ml, the number of aliquots was one to two, and the maximum amount of glue in each aliquot was between 2 and 3 ml. The calculated relationship between CVD and G_Total _ranged was CVD minus 0.5 to 1. Clinical and technical success was achieved in all. Two patients had intra-procedural, self-subsiding bleeding, and one patient had severe abdominal pain, which subsided with analgesics. None of them had fatal complications, transfusion requirements, or prolonged hospital stays.

Conclusions

CVD is a potential determinant factor for the total amount of glue injected during the endotherapy of GVs to achieve favorable clinical and technical outcomes.

## Introduction

Gastric varices (GVs) are common findings in upper gastrointestinal endoscopy (UGIE). They are reported in around 20% (5- 33%) of patients with portal hypertension either alone or with esophageal varices (EVs). About 22% of cirrhotics, 39% of extrahepatic portal vein obstruction, 30% of noncirrhotic portal fibrosis, and 6% of hepatic venous outflow obstruction (Budd-Chiari syndrome) may have gastric varices [1,2]. GVs bleed less commonly than EVs but the upper gastrointestinal bleed (UGIB) caused by GVs is usually massive and associated with high mortality [2]. Cyanoacrylate glue (CAG) injection, guided by either UGIE or endoscopic ultrasound (EUS), has become the standard of care for acute bleeding and secondary prophylaxis of high-risk GVs [3,4]. Although its role in primary prophylaxis is not well-defined, a few studies have also used CAG for this indication [5,6]. A CAG injection is a technically and clinically successful procedure, especially in experienced hands. The amount of glue injected is one of the most important determinant factors in the success as well as complications of the procedure. A smaller amount may fail to completely obliterate the GV, and larger amounts may lead to fatal complications, e.g., pulmonary embolization [7,8]. There are two methods of injecting CAG, first in smaller aliquots and second in larger aliquots. Experts and good-quality studies have recommended the use of a maximum of 4 ml CAG, in 0.5 ml aliquots to avoid complications [7,9]. Smaller aliquots require repeated endoscope insertion and use of multiple injector needles that may result in patient discomfort, prolongation of procedure time, endoscope damage, and increased cost. Few studies have used larger aliquots [9]. The current literature does not provide any clue for the objective determination of an accurate amount of glue for the safe and effective obliteration of GV pre-procedurally. Although missing in the published literature, an endoscopist imagines that GVs are either hemispherical or a mixture of hemispheres, hemicylinders, and various other geometrical shapes, and diameter or length is the most basic measurement to calculate their volume. The total amount of CAG may crudely correlate with the cumulative diameter of the varices (the sum of the maximum diameter of each varix if there are multiple varices). Our study aims to show that the variceal diameter is an objective parameter for deciding a safe and effective total volume of CAG to be injected. Our primary objective was to study technical success, and our secondary objectives were to study clinical success and complications.

## Materials and methods

Study design, setting, and duration

It is a retrospective analysis of endoscopy laboratory data of interventional cases. Institutional review clearance was taken (699/Ethics/2023). The study was performed after considering all parameters of good clinical practice and in accordance with ethical issues discussed in the Helsinki Declaration of 1975. Written, informed consent was taken for the procedure from each patient as a routine practice. The study was performed at the Gandhi Ward Endoscopy Laboratory of the Department of Medicine of King George’s Medical University, Lucknow. The study retrieved data of patients undergoing endoscopy between January 2022 and December 2022.

Study sample

The study retrieved data from patients who had bulbous GVs on UGIE and consented to undergo CAG injection. Since this was a retrospective case series, sample size calculation was not done, and all eligible patients during the study period were included.

Intervention

**Figure 1:**
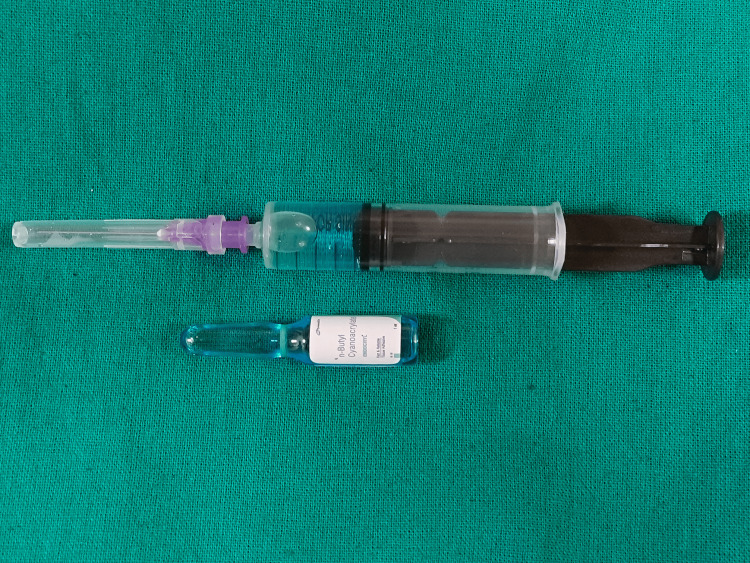
A single aliquot of cyanoacrylate glue

The calculated volume of CAG, based on CVD, was injected and divided into variable aliquot sizes (Figure 1), depending on the sizes and numbers of GVs.

Data collection

Demographic, clinical, and endoscopy data are routinely recorded on the case sheets, endoscopy charts, and videos of our laboratory software. For this study, we specially recorded the number, type (combined Sarin and Hashizume classification) [1,10,11], and size of GVs, total amount (GTotal) and aliquot sizes of CAG, and outcomes (technical success, intra-procedural, post-procedural complications) in each patient on a separate proforma, retrospectively reviewing the above records.

Definitions and standard operating procedure

Only large bulbous GV (F2 and F3) were treated with CAG injections. Technical success (GV obturation) was defined as firm non-collapsible varices on probing with an injector during the index endoscopy and after two weeks of follow-up. Clinical success was defined as the control of bleeding during the index procedure and the absence of rebleed on follow-up.

The technique of CAG was similar to that described by Kumar et al. [9], except that we injected a larger volume of CAG per aliquot, depending on the cumulative variceal diameter (CVD), as described later. After the CAG injection, patients were monitored in the post-procedure room or ward for the development of any complications by clinical assessment and vitals monitoring.

**Figure 2:**
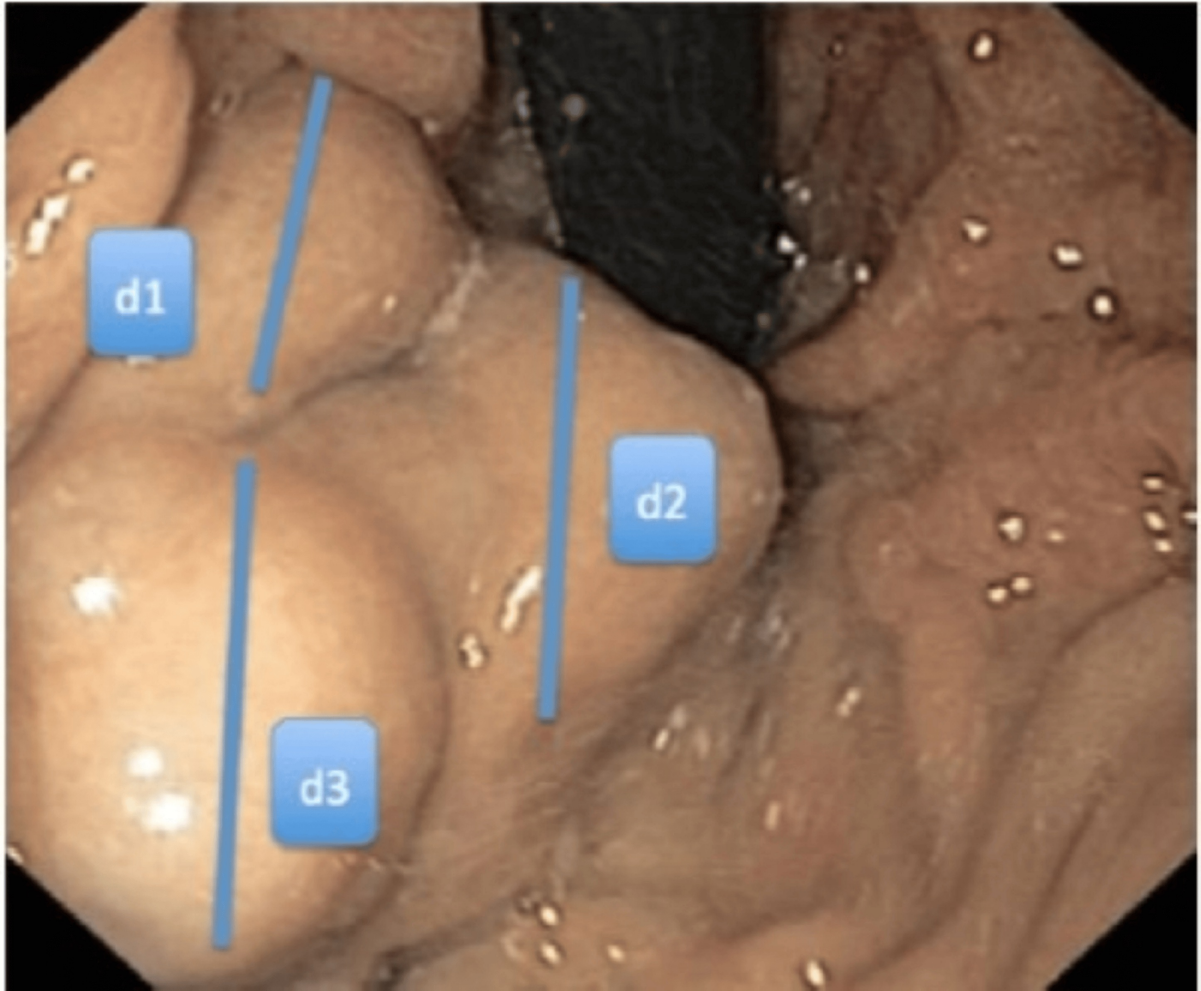
Calculation of cumulative variceal diameter (CVD) CVD is the sum of the maximum diameters of individual varices, CVD=d1+d2+d3

Videos of endoscopy were reviewed, and if needed, extra photographs were taken. CVD was calculated by summing the maximum diameters of all varices in each patient as shown in Figure 2. The maximum diameter of each varix was estimated by comparing it with the diameter of the endoscope in the fully inflated state of the stomach. The relationship between CVD and the total amount of CAG was calculated.

Statistical analysis

Frequency data were expressed as number, percentage, ratio, and range, as and when appropriate. The relationship between CVD and the total amount of CAG was calculated by simple subtraction.

## Results

A total of 467 UGI endoscopies were performed during the study period. Among them, 192 (41%) were cirrhotics and 275 (59%) non-cirrhotics. Eighteen patients had GVs, which was 4% of total UGI endoscopies and 9% of all cirrhotics. Six patients (1% of total UGI endoscopies) underwent a CAG injection for GVs. Among them, five had concomitant esophageal varices, four had a history of a recent upper gastrointestinal bleed, but none had an ongoing or acute bleed. The findings of our study are summarized in Table 1.

**Table 1:** Demographic, clinical, and endoscopic findings of patients undergoing cyanoacrylate glue injection and establishment of a relationship between cumulative variceal diameter and total amount of glue M: Male, F: Female, GV: Gastric varix, CVD: Cumulative variceal diameter (CVD was retrospectively calculated by summing the maximum diameter of each varix), cm: Centimeter, CAG: Cyanoacrylate glue, GTotal: Total amount of CAG injected, Y: Yes, N: No, IGVF and GOVF: Combined Sarin and Hoshizume classification of isolated gastric varices (IGV) and gastroesophageal varices (GOV)

S. N.	Variable	Overall summary (Ratio/Range/Description)	Case 1	Case 2	Case 3	Case 4	Case 5	Case 6
1.	Age (years)	18-68	34	68	52	65	55	18
2.	Sex (M: F)	5:1	M	M	F	M	M	M
3.	Type of GV	Classification	GOV2 F3	GOV2 F3	IGV1 F2/ F3	GOV2 F2/ F3	GOV2F3	GOV2F3
4.	Number of GV	1-4	3	3	3	4	3	1
5.	Size of each GV (cm)	0.5-2.5	1.0 +2.0 +2.0	1.0 +1.0 +2.0	0.5 +1.0 + 2.0	1.0 +1.0 +2.0	1.0 +1.0 +2.5	2.5
6.	CVD (cm)	3.5-5.0	5.0	4.0	3.5	4.0	5	2.5
7.	Total amount of CAG (ml)	2-4	4	3	3	3	4	2
8.	Aliquots (Number)	1-2	2	1	2	2	2	1
9.	Aliquot size (ml)	2-3	2	3	2	2	2	2
10.	Relation between CVD and G_Total_	G_Total_ = CVD- (0.5 to 1)	G_Total 1_ = CVD-1= 4	G_Total 2_ = CVD-1= 3	G_Total 3_ = CVD-0.5= 3	G_Total 4_ = CVD-1 = 3	G_Total 5_ = CVD-0.5 = 4	G_Total 6_ = CVD-0.5 = 2
11.	Technical success (Y:N)	All	Yes	Yes	Yes	Yes	Yes	Yes
12.	Complications	-	None	Abdominal pain	Moderate bleed	None	1. Needle stuck up, 2. Glue spillage	Massive bleed

The ages of the patients ranged from 18 to 68 years. Five were males, and one was female. Five patients had type 2 gastroesophageal varices (GOV2) (four F3 and one mix F2/F3 subtype) and one had type 1 isolated gastric varices (IGV1) of the F3 subtype. The observed relationship between CVD and the total amount of glue was CVD in centimeters minus 0.5 to 1 (Table 1).

One patient, in whom all the three observed varices were connected and a 3 ml size single aliquot of glue was used, developed severe abdominal pain and needed intramuscular diclofenac injection for pain relief. Two other patients developed a moderate to massive intra-procedural bleed, which settled after 10-15 seconds spontaneously. In both, the bleed was neither fatal nor required transfusion or prolongation of hospital stay.

## Discussion

Ours is probably the first study to describe the preprocedural, objective assessment of the total amount of glue injected in a single patient. The hustle of a mid-procedure shortage of CAG or post-procedure storage of syringe-filled CAG (which later on gets destroyed) is a commonly faced problem, apart from safety and efficacy-related issues, if a preprocedural assessment of the total amount of glue to be injected is not done.

Our study produced the optimal desired result, i.e. a simple formula establishing a relationship between CVD and the total amount of glue to be injected in GVs. Besides it showed glimpses of the prevalence of GVs in our endoscopy laboratory, the frequency of the glue injection procedure, clinico-technical success, and the complications and limitations of our setup. In our study, GVs were seen in 4% of total UGI endoscopies and 9% of all cirrhotics, which was smaller than in other studies. In the literature, about one-fifth of cirrhotics are reported to have GVs [1].

CAG has become the standard of care to treat GVs [3,4]. Most studies have described the use of CAG in 0.5 ml aliquots of the undiluted CAG or 1.2-1.5 ml aliquots of the mixture of 0.5 ml CAG plus 0.7-1.0 ml lipiodol [12-14]. In the study by Sarin et al., the total volume used in a single varix varied between 1.2 and 4.6 ml and the total volume used per patient was 3.6+1.3 ml [12]. One study using undiluted CAG in 0.5-1.0 ml aliquots described the maximum amount of CAG to be 4 mL in 1 session and 2 ml per varix depending on the size [9]. In these studies, the volume of glue assessment was not based on preprocedural objective measurement of the size of the varix. In our study, we used aliquot sizes of a minimum of 2 ml and a maximum of 3 ml. The 2 ml aliquot was safe and effective in all cases. The 3 ml aliquot was effective but resulted in severe abdominal pain in one patient, who needed intramuscular diclofenac injection. Although no further complication occurred in this patient, amounts of CAG larger than 2 ml in a single aliquot should be avoided.

In search of accuracy and ease, EUS-guided glue injection, coiling, or a combination and angiography-guided obliteration of GVs (balloon-occluded retrograde transvenous obliteration, BRTO; balloon-occluded antegrade transvenous obliteration, BATO; plug-assisted antegrade transvenous obliteration, PATO, etc.) evolved in due course of time [14-18]. However, the availability and expertise of these advanced technologies may not be available, and/or various other constraints (e.g. clinical and financial) may not allow the use of these modalities in all patients at all centers. So the importance of UGIE-guided glue injection and CVD cannot be underestimated.

Whenever possible, an EUS-guided coil plus glue combination should be used for GVs. However, in the absence of these advanced facilities, the CVD may guide the total amount of glue to be injected. Since the amount of glue determines the technical and clinical success as well as the development of complications, neither too small nor too large an amount of glue should be used. Even if the calculated total amount exceeds 4 ml, the injected total amount should be limited to 4 ml only. If the CVD exceeds 4 cm, the subtraction integer should be 1, and if the CVD is less than 4 cm, the subtraction can be 0.5. Besides the aliquot size of 3 ml produced severe abdominal pain in our one case and other serious complications have been reported [7].

Strengths and limitations

The study has several strengths. First, it is a pioneering work in using an objective, preprocedural measure (CVD) to determine the amount of glue to be injected. The study's formula was simple and practical, showing the relationship between CVD and glue volume, which can be applied in busy endoscopy units. It also provided important clinical insights into the prevalence of GVs and procedural success in the study setting. Moreover, the 2 ml aliquot size was found to be safe and effective in all cases, which is an important finding for clinical practice.

However, the study also had notable limitations. It was a single-center study with a small sample size, limiting the generalizability and statistical significance of its findings. There was no predefined criterion for the total amount of glue, and the relationship between CVD and glue volume was derived retrospectively from earlier cases, raising concerns about the robustness of the method. The study only included type 2 GOV and type 1 IGV varices, potentially limiting its applicability to other types of GVs. Additionally, while the CVD-based formula is practical, it may oversimplify the true volume of the varix, as other factors (such as afferent and efferent veins) are also important. Subtracting 0.5-1.0 ml from the calculated volume was suggested to overcome this limitation. The use of larger aliquots (3 ml) caused severe abdominal pain in one patient, and complications from larger aliquots have been reported in other studies as well. Multicenter, well-designed studies with larger sample sizes are needed to validate these findings and compare them to more advanced techniques such as EUS-guided glue injections and angiographic methods.

Clinical implications

The study's findings have practical implications for the clinical management of GVs. In the absence of advanced technologies, such as EUS-guided glue injection or angiographic methods like BRTO or BATO, the CVD-based formula for determining glue volume can be a valuable tool. Since the amount of glue used is crucial for technical and clinical success, care must be taken to avoid using either too little or too much glue. The study recommends limiting the total injected volume to 4 ml, even if the calculated total exceeds this value. The formula also suggests that if the CVD is greater than 4 cm, a subtraction factor of 1 should be applied, and if it is less than 4 cm, a subtraction of 0.5 is recommended. In cases where advanced technologies are unavailable, this simplified method offers a feasible solution.

## Conclusions

To conclude, about 1% of all UGIEs need glue injection for GVs. UGIE-guided glue injection is safe in experienced hands. The approximate amount of total glue (in milliliters) to be injected, was calculated by subtracting 0.5 to 1 from the cumulative diameter of GVs (in centimeters), which is the sum of the maximum diameter of individual varices. Aliquot sizes of 1-2 milliliter glue can be used safely. More than 2 milliliter aliquots may be associated with complications.
